# Supplementary material for: Olfactory Choice for Decomposition Stage in the Burying Beetle Nicrophorus vespilloides: Preference or Aversion?
Source: Insects. 2020 Dec 26;12(1):11. doi: 10.3390/insects12010011 (PMC7824017; doi:10.3390/insects12010011)
Supplement: Supplementary file 1 [file insects-12-00011-s001.zip › Delclos et al Insects 2020 Supplementary Materials.docx]

**Supplementary Materials**

**
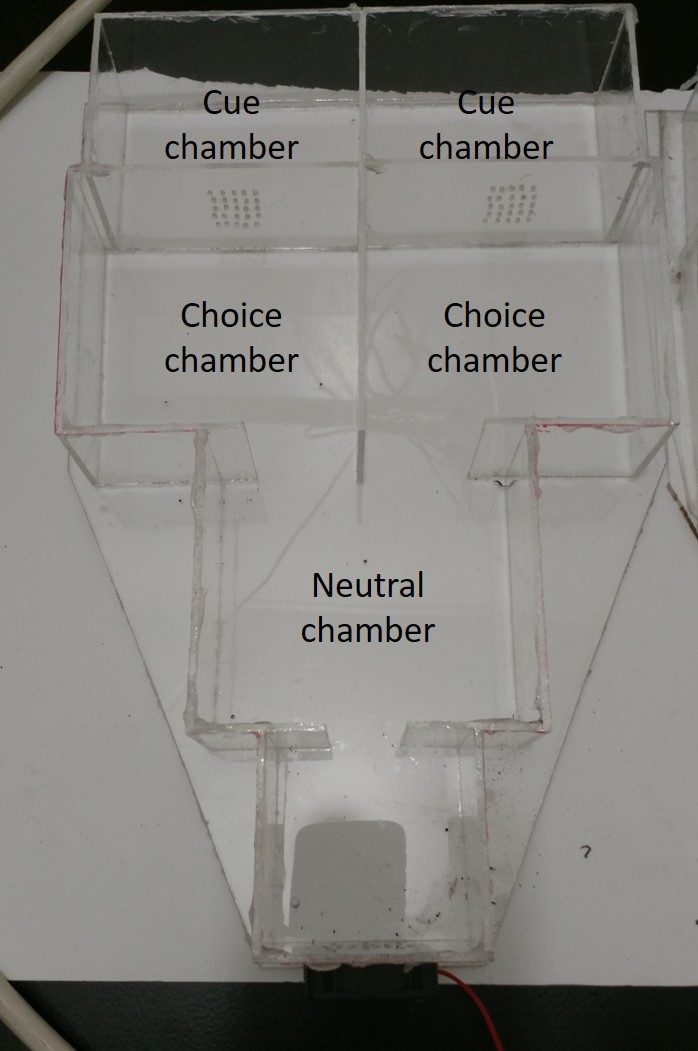
**

**Figure S1 –** Arenas used for assaying olfactory preferences of mated female *Nicrophorus vespilloides*. Arenas are covered with a clear acrylic lid, fitted with two charcoal filters that allow filtered air to enter through the cue chambers and exit via a USB-powered fan attached to the neutral chambers (bottom of picture).

**
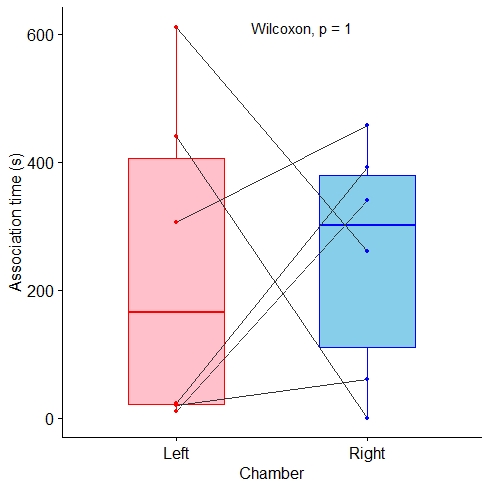
**

**Figure S2 –** Negative control olfactory preference assays testing for side biases in mated female *Nicrophorus vespilloides*. Both chambers (left and right) received cues from an empty choice chamber. A total of twelve mated female beetles were tested (six responsive). Data points refer to association times of individual females, and boxplots denote median values and upper- and lower-quartiles.

**
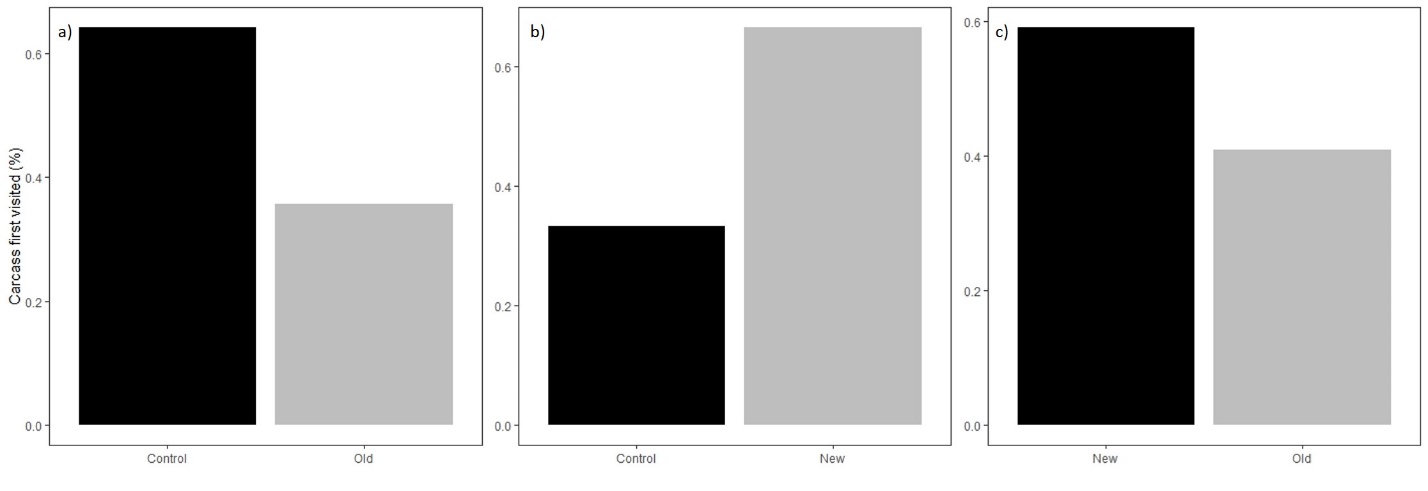
**

**Figure S3 –** Percentage of females first visiting: a) an empty (Control) choice chamber or choice chamber receiving 7-day-old (Old) carcass cues, b) an empty (Control) choice chamber or chamber receiving 1-day-old (Fresh) carcass cues, and c) a choice chamber containing fresh or old carcass cues.
